# Effects of low-molecular-weight chitosan on the growth performance, intestinal morphology, barrier function, cytokine expression and antioxidant system of weaned piglets

**DOI:** 10.1186/s12917-018-1543-8

**Published:** 2018-07-04

**Authors:** Shenglan Hu, Yu Wang, Xiaolu Wen, Li Wang, Zongyong Jiang, Chuntian Zheng

**Affiliations:** 1 Institute of Animal Science, Guangdong Academy of Agricultural Sciences; State Key Laboratory of Livestock and Poultry Breeding; Key Laboratory of Animal Nutrition and Feed Science in South China, Ministry of Agriculture; Guangdong Public Laboratory of Animal Breeding and Nutrition; Guangdong Key Laboratory of Animal Breeding and Nutrition, #1 Dafeng 1st Street, Wushan, Tianhe District, 510640 Guangzhou, Guangdong People’s Republic of China; 2 Hebei depond animal health care science and technology co., Ltd, #8 shuangtong Road, Mengtong, Luquan District, 050204 Shijiazhuang, Hebei People’s Republic of China

**Keywords:** Low-molecular-weight chitosan, Weaned piglet, Growth performance, Immune response, Intestinal barrier function

## Abstract

**Background:**

Chitosan was used as an alternative to promote the growth of weaned piglets. And low-molecular-weight chitosan (LC) is one of chitosan derivatives and maintain beneficial biological properties of chitoson. The present experiment was carried out to examine the effects of LC on the growth performance, intestinal morphology, barrier function, cytokine expression, and antioxidant system of weaned piglets.

**Results:**

A total of 40 piglets weaned at 21 d of age, with average body weight 6.37 ± 0.08 kg, were randomly assigned (5 pens/diet; 4 pigs/pen) to 2 treatments (a basal diet and the basal diet supplemented with 50 mg/kg LC) and were fed for 28 d. Compared with the control group, average daily feed intake (ADFI), and the expression of intestinal barrier protein *ZO-*1 was increased (*P* < 0.05) when the piglets fed the diet supplemented with LC. No significant differences were found in average daily gain (ADG, *P* > 0.05), gain-to-feed ratio (G:F, *P* > 0.05), the incidence of diarrhea (*P* > 0.05), or the antioxidant capacity (*P* > 0.05) between two groups. The expression of *IL-1β* and *TNF-α* in jejunal mucosa were significantly decreased (*P* < 0.05) in piglets fed the LC-supplemented diet in comparison to the control.

**Conclusion:**

The results of this study indicate that dietary supplementation with LC at 50 mg/kg was effective for enhancing the growth performance in weaned piglets, improving intestinal barrier function and alleviating intestinal inflammation.

## Background

Weaning is an important stage of gut development and may cause low feed intake, intestinal dysfunction and growth retardation in piglets [1, 2]. Antibiotics have long been used to solve problems in the weaning period and to promote the growth and health of piglets [3]. As the use of antibiotics can lead to bacterial resistance and potential antibiotic residues in animal products, thus many alternatives to antibiotics have been suggested and tested. Chitosan with the molecular weight of 1000 kDa, is the second most abundant carbohydrate polymer in nature [4, 5]. It has been reported that chitosan has been widely used as a potential alternative to in-feed antibiotics in piglets and broiler chickens due to many beneficial biological properties of it, such as promoting the growth performance [6, 7], anti-oxidative [8] and immunity modulation [9]. However, chitosan is restricted to be used in food and biomedical applications because of its difficult solubility and instability [10]. Low-molecular-weight chitosan (LC, < 150 kDa) and chito-oligosaccharide (COS, < 5 kDa) are obtained from chitosan by physical, chemical or enzymatic methods, and have much higher solubility and stability than chitosan. Chito-oligosaccharide (COS) with the properties of antimicrobial, anti-inflammatory, anti-oxidative and immunity modulate [11, 12], is widely used as a dietary additive in livestock. But it is still unclear whether dietary supplementation with LC can affect the piglets. The objective of the present experiment, therefore, was to clarify the effects of low-molecular-weight chitosan on the growth performance, incidence of diarrhea, intestinal morphology, barrier function, immune response, and antioxidant system in weaned piglets.

## Methods

### Ethics statements

The piglets examined in the present study were approved by the Animal Care and Use Committee of the Institute of Animal Science, Guangdong Academy of Agricultural Sciences, with the approval number of GAASISA-2016-017.

### Animals and experimental treatments

A total of 40 Duroc × Landrace × Yorkshire piglets weaned at 21 d of age were blocked by BW (average 6.37 ± 0.08 kg), and randomly assigned to 2 treatments with 4 pigs per pen and 5 replicate pens per treatment. The piglets were purchased from WanHe Nongmu Co., Ltd., Guangdong, China. These were (1) a control group (CON) fed the basal diet, and (2) the basal diet supplemented with 50 mg/kg low-molecular-weight chitosan (LC); both were fed for 28 d. The LC (molecular weight 20 to 30 kDa), which was obtained from chitosan by radiation pyrolysis technology, was offered by Jiaxing Korui Biotech Co., Ltd., Zhejiang, China. The composition and content of the treatment diets were shown in Table 1. Piglets were housed in a temperature-controlled nursery and had ad libitum access to feed and water. The body weight of piglets and amount of the feed was measured to calculate the average daily gain (ADG), average daily feed intake (ADFI), and gain-to-feed ratio (G:F). The number of pigs with diarrhea was recorded every day. Diarrhea index (%) was calculated as 100 × number of piglets that had diarrhea/total number of piglets.

**Table 1:** Ingredient and chemical composition of the basal diet (as-fed basis)

Ingredient (%)	Control	LC
Corn	60.43	60.43
Extruded soybean meal	20.00	20.00
Fish meal	5.00	5.00
Soybean protein concentrate	2.00	2.00
Whey powder	7.50	7.50
Soybean oil	1.50	1.50
L-Lysine.HCl	0.25	0.25
DL-Met	0.10	0.10
CaHPO_4_	1.05	1.05
Limestone	0.75	0.75
50% Choline Chloride	0.12	0.12
NaCl	0.30	0.30
Premix^a^	1.00	1.00
Low-molecular-weight chitosan	0.00	0.005
Calculated analysis		
DE, MJ/kg	14.10	14.10
CP (%)	19.55	19.55
Lys (%)	1.17	1.17
Met (%)	0.50	0.50
Met+Cys (%)	0.74	0.74
Thr (%)	0.77	0.77
Ca (%)	0.86	0.86
Available P (%)	0.68	0.68

^a^Premix supplied per kg: 11000 IU of vitamin A; 1100 IU of vitamin D_3_; 80 IU of vitamin E; 2.5 mg of vitamin K_3_; 17.5 mg of vitamin B; 20 mg of vitamin B_2_; 10 mg of vitamin B_6_; 220μg of vitamin B_12_; 150 mg of nicotinamide; 1.5 mg of D-calcium pantothenate; 1.5 mg of folic acid; 3 mg of biotin; 150 mg of Fe (FeSO_4_); 10 mg of Cu (CuSO_4_); 10 mg of Mn (MnSO_4_); 150 mg of Zn (ZnO); 0.2 mg of I (KIO_3_); 0.3 mg of Se (Na_2_SeO_3_) and 0.15 mg of Co (LCO_4_)

### Sample collection

The BW of each piglet was recorded at the end of the experiment. After 12 h fasting, five pigs (1 per pen) were randomly selected from each treatment and anaesthetised with pentobarbital sodium (50 mg/kg, i.v.). After sedation, serum was obtained by centrifuging at 2000×*g* for 10 min and stored at − 20 °C for subsequent measurements of activities of catalase (CAT), glutathione peroxidase (GSH-Px), and total superoxide dismutase (T-SOD), and contents of malondialdehyde (MDA) and total antioxidant capacity (T-AOC), using commercial kits (Nanjing Jiancheng Bioengineering Institute, Nanjing, China). The gastrointestinal tract was immediately removed after slaughter, washed with cold PBS. And then 2–3 cm segments of duodenum, jejunum and ileum were removed and fixed in 4% formaldehyde for histometric analysis. Mucosa was scraped from a 10–15 cm segment of jejunum and stored at − 80 °C for gene expression analyses.

### Analysis of small intestinal morphology

The fixed intestinal segments were dehydrated and embedded using low-melt paraffin wax by routine methods. Three cross-sections (5 μm) of each segment were dewaxed then stained with hematoxylin and eosin. Villus height and crypt depth of each intestinal segment were measured at 10 × magnification using an image processing and analysis system. At least 10 well-oriented intact villi and their associated crypts were examined in each intestinal segment of each piglet. The mean villus height and crypt depth of each section were calculated per piglet and used for further analysis.

### Relative quantification of mRNA expression

Total RNA from jejuna mucosa was extracted according to the manufacturer’s instructions of TRIzol reagent (Invitrogen, Carlsbad, CA). The concentration of RNA was measured by NanoDrop ND-1000 (Thermo Fisher Scientific). And the integrity of RNA was checked by electrophoresis on 1% agarose gel. After removed genomic DNA with gDNA Eraser (TaKaRa, Dalian, reverse transcription was carried out using PrimeScript™RT reagent kit (TaKaRa, Dalian, China) followed the manufacturer’s instructions. The expression levels of intestinal tight junction and cytokines were analyzed using SYBR® Premix Ex Taq™ II kit (Takara, Dalian, China) and iQ™5 Real Time PCR Detection System (Bio-Rad, Hercules, CA, USA). The sequences of the primers were listed in Table 2. The 2^-ΔΔCt^ method was used to estimate the abundance of mRNA.

**Table 2:** Primer sequences for real-time PCR analysis

Gene	Sequence(5′ → 3′)	Product size (bp)	GenBank accession number
*ZO-1*	CTCTTGGCTTGCTATTCG	256	XM_003353439.2
	AGTCTTCCCTGCTCTTGC		
*Occludin*	GTAGTCGGGTTCGTTTCC	167	NM_001163647.2
	GACCTGATTGCCTAGAGTGT		
*IL-1β*	CTCCAGCCAGTCTTCATTGTTC	132	NM_214055.1
	TGCCTGATGCTCTTGTTCCA		
*TNF-α*	CACCACGCTCTTCTGCCTAC	116	X54859
	ACGGGCTTATCTGAGGTTTGAGACG		
*IL-10*	GGTTGCCAAGCCTTGTCAG	202	NM_214041
	AGGCACTCTTCACCTCCTC		
*TGF-β*	GAAGCGCATCGAGGCCATTC	162	NM_214015
	GGCTCCGGTTCGACACTTTC		
*β-actin*	CGGGACATCAAGGAGAAGC	273	DQ845171
	ACAGCACCGTGTTGGCGTAGAG		

### Statistical analysis

All data were subjected to a t-tests using SAS (Version 8.1; SAS Inst. Inc., Cary, NC). Data are presented as means and SEM. *P*-values < 0.05 were used to indicate statistical significance.

## Results

### Growth performance and incidence of diarrhea

As shown in Table 3, ADG and ADFI was increased by 22.28% (*P* = 0.084) and 8.95% (*P* < 0.05) in the piglets fed with dietary supplementation with low-molecular-weight chitosan (LC). No significant changes were found in G:F and rate diarrhea rate between control group and LC group.

**Table 3:** Effect of dietary supplementation with LC on growth performance of weaned piglets

Variables	CON	LC	SEM	*P*-value
ADG (g/d)	177.7	217.3	10.03	0.084
ADFI (g/d)	297.3^b^	323.9^a^	9.93	0.013
G:F (g/g)	0.63	0.66	0.02	0.391
Diarrhea rate (%)	26.32	25.97	1.08	0.882

*ADG* average daily gain, *ADFI* average daily feed intake, *G:F* gain-to-feed ratio, *CON* piglets fed the basal diet, *LC* piglets fed the basal diet with 50 mg/kg 20–30 kDa chito-oligosaccharide, *SEM* standard error of mean

^a,b^Values within a row with different superscripts differ significantly at *P* < 0.05. *n* = 5

### Small intestinal morphology

**Fig. 1:**
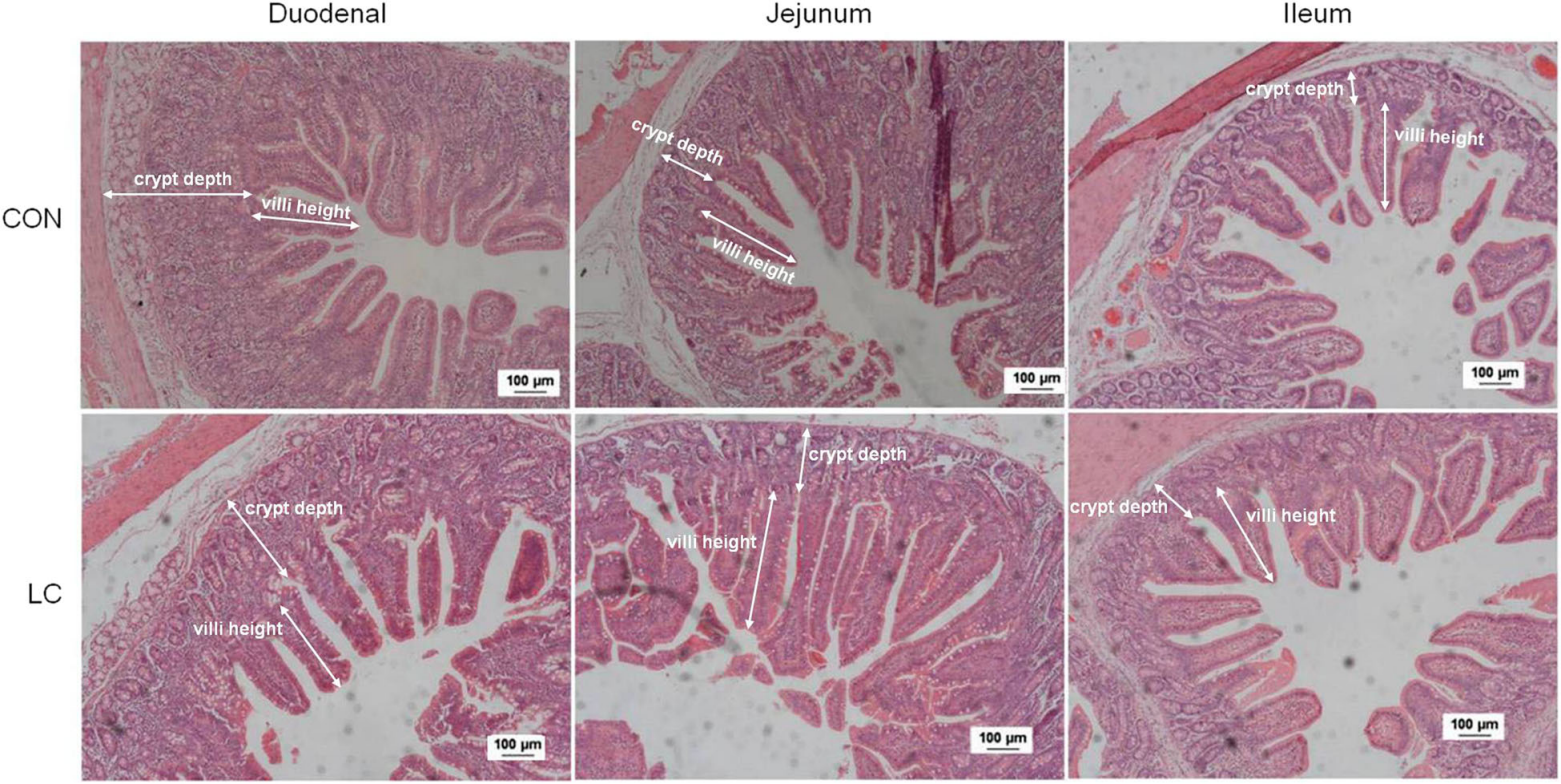
Effects of LC on intestinal morphology of duodenum, jejunum and ileum. CON: piglets fed the basal diet; LC: piglets fed the basal diet with 50 mg/kg 20–30 kDa low-molecular-weight chitosan. The scale bars in each image indicate 100 μm

No significant differences in villus height, crypt depth, or villus height:crypt depth in duodenum, jejunum and ileum were observed between CON and LC groups (Table 4, Fig. 1).

**Table 4:** Effect of LC supplementation on morphology of the small intestine of weaned piglets

Group	Villus heigh (μm)			Crypt depth (μm)			VH:CD (μm: μm)		
	Duodenal	Jejunum	Ileum	Duodenal	Jejunum	Ileum	Duodenal	Jejunum	Ileum
CON	376.0	375.1	330.8	337.1	245.2	182.8	1.16	1.57	1.88
LC	424.0	373.8	330.7	295.9	218.4	213.1	1.45	1.71	1.58
SEM	15.22	20.81	0.09	9.83	10.74	0.07	14.34	13.98	0.12
*P*-value	0.118	0.351	0.106	0.952	0.232	0.380	0.768	0.349	0.247

*CON* piglets fed the basal diet, *LC* piglets fed the basal diet with 50 mg/kg 20–30 kDa low-molecular-weight chitosan, *VH:CD* villus height: crypt depth, *SEM* standard error of mean, n = 5

### Intestinal cytokines

**Fig. 2:**
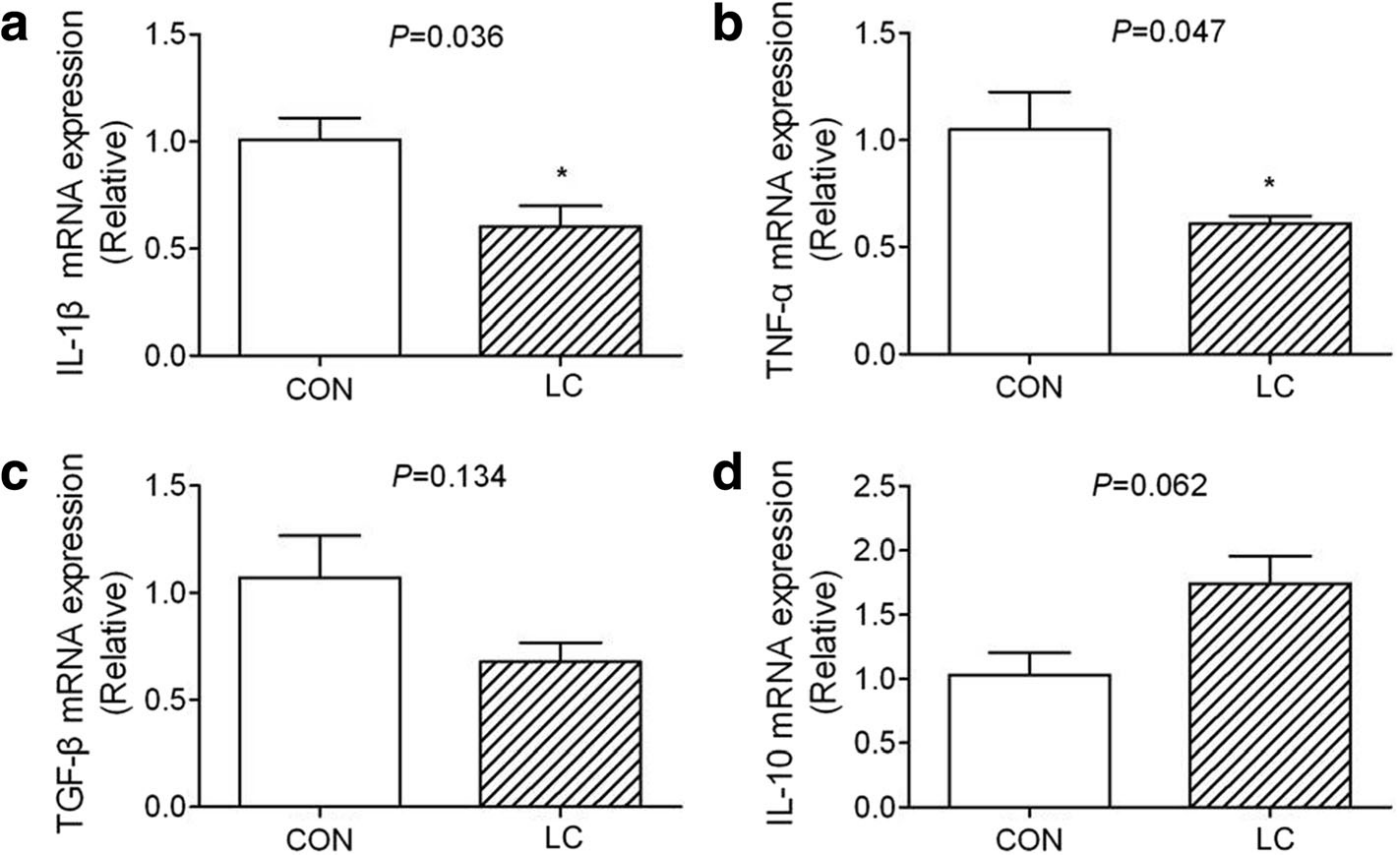
The pro-inflammatory cytokine gene expression was inhibited by LC**.** *IL-1β* (**a**), *TNF-α* (**b**), *TGF-β*(**c**) and *IL-10* (**d**) were quantified by RT-PCR. CON: piglets fed the basal diet; LC: piglets fed the basal diet with 50 mg/kg 20–30 kDa low-molecular-weight chitosan. Five piglets per treatment. *P < 0.05

Dietary supplementation with low-molecular-weight chitosan (LC) significantly decreased (*P* < 0.05) the expression of *IL-1β* and *TNF-α* in the jejunal mucosa of piglets in comparison to the CON group (Fig. 2a-b). There were no significant differences in the jejunal mucosal expression of *TGF-β* and *IL-10* (Fig. 2c-d).

### Anti-oxidation indices

The effect of LC on the activity of serum antioxidant enzymes and indices in weaned piglets are shown in Table 5. While there were no significant differences, activities of antioxidant enzymes, T-AOC, CAT, GSH-Px and T-SOD were slightly higher in the piglets fed LC-supplemented diets, and the concentration of MDA in serum was reduced.

**Table 5:** Effect of LC supplementation on serum anti-oxidation indices of weaned piglets

Group	CON	LC	SEM	*P*-value
CAT (U/mL)	101.3	107.3	7.13	0.704
GSH-Px (U/mL)	44.1	45.4	0.69	0.357
T-SOD (U/mL)	111.6	114.2	4.17	0.779
MDA (nmol/mL)	3.10	1.74	0.55	0.233
T-AOC (U/mL)	1.45	1.82	0.10	0.064

*CON* piglets fed the basal diet, *LC* piglets fed the basal diet with 50 mg/kg 20–30 kDa low-molecular-weight chitosan, *CAT* activities of catalase, *GSH-Px* glutathione peroxidase, *T-SOD* total superoxide dismutase, *MDA* contents of malondialdehyde, *T-AOC* total antioxidant capacity, *SEM* standard error of mean, n = 5

### Indices of intestinal barrier function

**Fig. 3:**
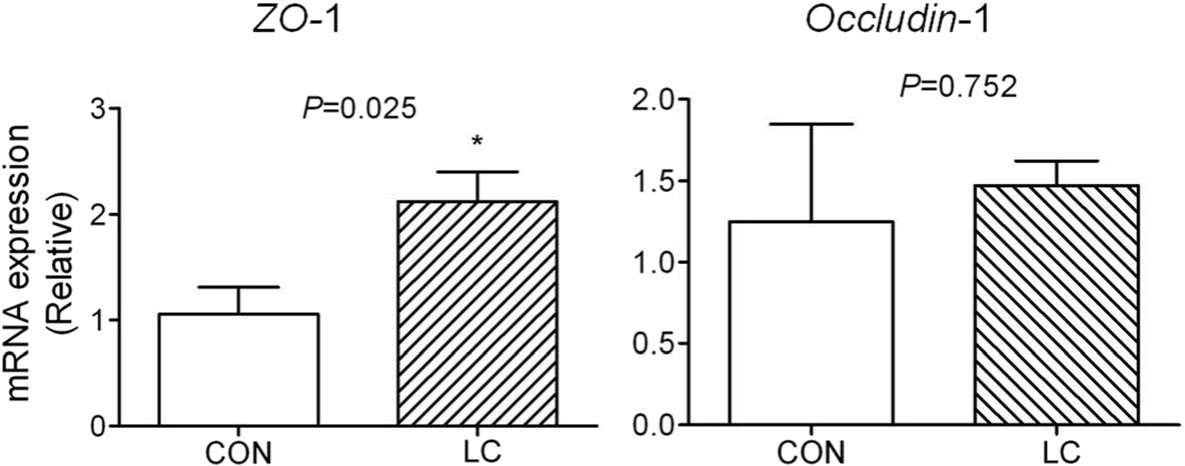
Effect of dietary LC on jejunal expression of intestinal barrier genes. CON: piglets fed the basal diet; LC: piglets fed the basal diet with 50 mg/kg 20–30 kDa low-molecular-weight chitosan. Five piglets per treatment. *P < 0.05

Compared with the piglets in control group, the relative expression of jejuna mucosa *ZO*-1 transcripts was dramatically increased (*P* = 0.025) in piglets from LC group, while no significant change was found in the expression of *Occludin*-1 (Fig. 3).

## Discussion

Chitosan, one of the most abundant polymers in nature, is an alkaline polysaccharide with positive charges [13]. Because of its characteristics of nontoxic [14], and the biological activities, such as antimicrobial activity and anti-inflammatory [15, 16], chitosan is widely used as a dietary supplement in livestock industry to promote the growth [13, 17]. However, the influences of low-molecular-weight chitosan (LC) on the growth of livestock remain unknown. In the present study, the effect of dietary supplementation with 50 mg/kg LC (20–30 kDa) on growth performance, incidence of diarrhea, intestinal morphology, intestinal barrier, cytokines expression and anti-oxidation indices were examined in weaned piglets. The results demonstrated that, after 4 wk. of post-weaning feeding, there were no significant differences in ADG and G:F between piglets fed the LC-containing and control diets. When compared with the piglets fed the CON diet, ADFI was significantly increased by about 9% and ADG increased 22% in the piglets fed LC. While the latter was not significant, it was of greater magnitude than the change in ADFI, suggesting that the effects of LC on nutrient metabolism might mediate part of the growth response. It has been demonstrated that low molecular weight chitosans prevented the increases in bodyweight by decreasing the absorption of dietary lipids [18] and intestinal disaccharidase activities [19]. Many studies showed positive effects of another small molecular weight COS on the growth performance of weaned pigs; other studies found that there were no significant differences between piglets fed COS-supplemented and CON diets [20, 21]. Walsh et al. [22] demonstrated that 5 to 10 kDa COS possessed antibacterial activity and the 10 to 50 kDa preparation was optimum for enhancing intestinal structure. It can be speculated that the different effects of the LC on growth performance of weaned piglets may result from the molecular weight, dosage, solubility or the duration of LC supplementation.

Diarrhea in the post-weaning period is always due to intestinal dysfunction. The decrease of villus height and the increase of crypt depth associated with dysfunction have been found in previous studies [23–25]. Liu et al. [20] observed that 160 mg/kg supplemental COS reduced the incidence of diarrhea and increased the villus:crypt ratio in weaned piglets challenged with *Escherichia coli*. Supplementation with 200, 400, or 600 mg/kg COS in diets of weaned piglets not influencing the villus:crypt ratio in duodenum, jejunum, or ileum [7]. In the results of the present experiment, basic dietary-supplemented with LC at 50 mg/kg did not influence incidence of diarrhea, villus height, crypt depth and villus:crypt ratio in the duodenum, jejunum and ileum. Supplementation with LC at 50 mg/kg tended to increase the villus:crypt ratio in duodenum and jejunum of the piglets. Tight junctions are the important determinants of epithelial barrier functions [26]. When the piglets feed with 30 mg/kg COS, the expression of epithelial tight junction, such as occluding-1 and ZO-1 was decreased [21]. The present study found that the jejuna mucosa expression of *ZO-1* was significantly enhanced by 50 mg/kg dietary LC (20–30 kDa), thought it did not affect the incidence of diarrhea in weaned piglets. Further researches were needed to be carried out to explore the effects of low dosage of COS on intestinal barrier function.

The balance between pro-inflammatory and anti-inflammatory cytokines is of great importance for the health of weaned piglets [27]. It has been reported that the intestinal expression of pro-inflammatory cytokine genes, such as *IL-1β*, *IL-6*, and *TNF-α*, is up-regulated in weaned piglets [28] and the present study found that dietary supplementation with 50 mg/kg LC (20–30 kDa) significantly reduced the jejunal mucosal expression of pro-inflammatory cytokines *IL-1β* and *TNF-α* while not influencing that of anti-inflammatory cytokines *IL-10* and *TGF-β*. The data suggest that dietary supplementation with 50 mg/kg LC modulated immune responsivity by inhibiting the expression of pro-inflammatory cytokines in weaned piglets.

Oxidative stress accompanies weaning and plays a very important role in intestinal health [29]. The present study found that dietary supplementation with 50 mg/kg LC tended to increase the concentration of the total anti-oxidant capacity and decrease the concentration of MDA in serum, while all the indices showed no significant difference. Previous study reported that dietary supplementation with 30 mg/kg COS had dramatically inhibited T-AOC and T-SOD [21]. In contrast, no significant differences in the antioxidant enzymes and MDA were observed here in piglets supplemented with 50 mg/kg LC. It is widely known that diarrhea usually activates the antioxidant system [30], so the very slight changes in activities of antioxidant enzyme and the concentration of MDA were consistent with the incidence of diarrhea compared the control and LC-groups.

## Conclusions

These observations suggested that 50 mg/kg low-molecular-weight chitosan (LC) supplements enhanced the growth of weaned piglets, improved intestinal barrier and inhibited intestinal inflammation. The findings will contribute to the guidance on LC supplements.

## Abbreviations

ADFI: Average daily feed intake
ADG: Average daily gain
BW: Body weight
CAT: Activities of catalase
CON: Control
COS: Chito-oligosaccharide
G:F: Gain-to-feed ratio
GSH-Px: Glutathione peroxidase
LC: Low-molecular-weight chitosan
MDA: Contents of malondialdehyde
T-AOC: Total antioxidant capacity
T-SOD: total superoxide dismutase
